# Supplementary material for: Heterodimerization of Mu Opioid Receptor Protomer with Dopamine D2 Receptor Modulates Agonist-Induced Internalization of Mu Opioid Receptor
Source: Biomolecules. 2019 Aug 14;9(8):368. doi: 10.3390/biom9080368 (PMC6722706; doi:10.3390/biom9080368)
Supplement: Supplementary file 1 [file biomolecules-09-00368-s001.pdf]

Supplementary information

Table S1: List of primers used in the study: Primers used for the development of the GPCR–NanoBiT® fusion constructs. a: Forward (F) and Reverse (R) primers (5′ →3′) with restriction enzyme sites (underlined). b: Annealing temperature. c: Restriction enzyme.

	Primers (5′ → 3′) [a]	Restriction site	Tm (°C)[b]	Fusion construct
F	TAG<u>AAGCTT</u>ATGGATCCACTGAATCTGTCC	*Hin*dIII, *Eco*RI	60	D_{2L}R-LgBiT
R	AGTATA<u>GAATTC</u>TTGCAGTGGAGGATCTTCAGG			
F	TAG<u>AAGCTT</u>ATGGATCCACTGAATCTGTCC	*Hin*dIII, *Eco*RI	60	D_{2L}R-SmBiT
R	AGTATA<u>GAATTC</u>TTGCAGTGGAGGATCTTCAGG			
F	TAG<u>AAGCTT</u>ATGGACAGCAGCGCTGCC	*Hin*dIII, *Xho*I	60	MOR-LgBiT
R	GTAG<u>CTCGAG</u>TTGGGCAACGGAGCAGTTTC			
F	TAG<u>AAGCTT</u>ATGGACAGCAGCGCTGCC	*Hin*dIII, *Xho*I	60	MOR-SmBiT
R	GTAG<u>CTCGAG</u>TTGGGCAACGGAGCAGTTTC			

Table S2: Overview of the functionality of constructs used in the study using various assays and agonists.

Construct	Dopamine	DAMGO	Assay used
D_{2L}R-EGFP	+	–	Receptor internalization
MOR-YFP	NA	+	Receptor internalization
D_{2L}R-LgBiT	+	NA	Calcium release
D_{2L}R-SmBiT	+	NA	Calcium release
MOR-LgBiT	NA	+	Calcium release
MOR-SmBiT	NA	+	Calcium release
Untransfected HeLa cells	–	–	Calcium release

HeLa cells were transfected with the above-mentioned constructs. After 48 h, functionality of the tagged receptors was evaluated by monitoring their capacity to internalize or stimulate calcium release upon stimulation with their respective agonists. "+": indicates calcium release or receptor internalization observed; "–": no change observed; NA: not analyzed.

Figure S1:

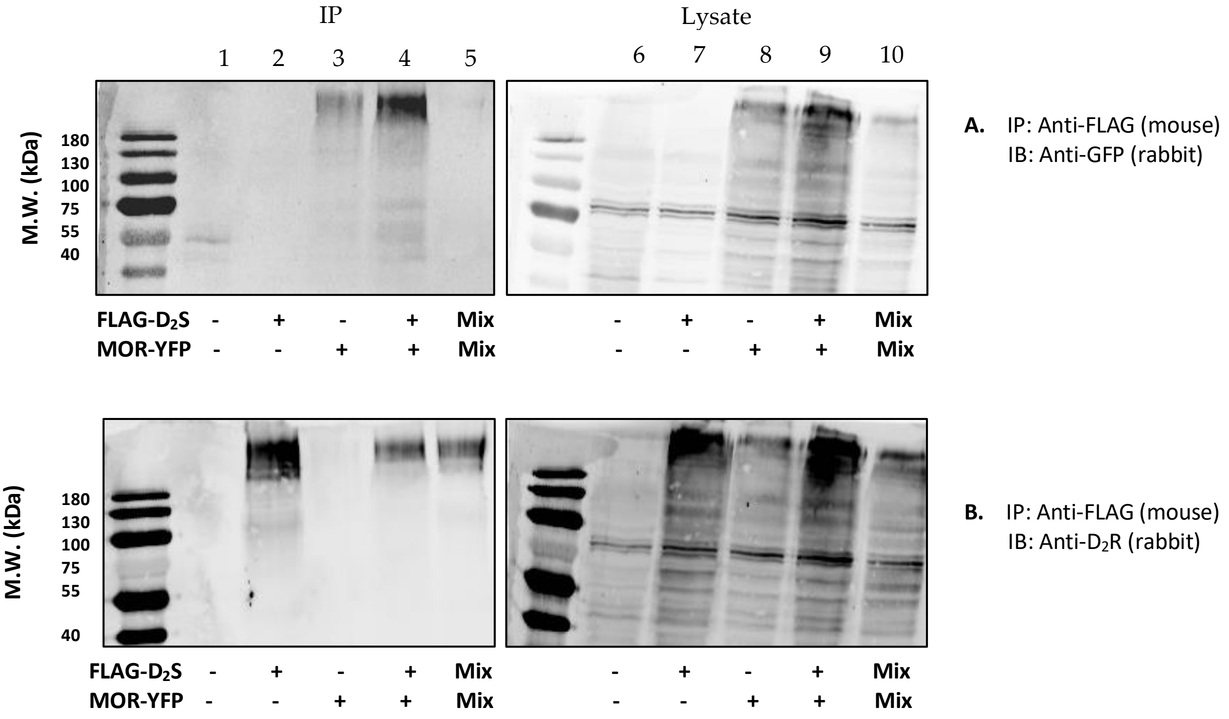

Figure S1: Analysis of $D_{2S}R$ receptor dimerization with MOR using co-immunoprecipitation. HEK 293T cells were transiently transfected with pFLAG-$D_{2S}R$, pMOR-YFP or both. After 48 h, the cells were lysed and an aliquot was loaded onto SDS-PAGE followed by immunoblotting with anti-D_2R or anti-GFP. The rest of the lysates was subjected to IP with anti-FLAG. Co-immunoprecipitation of $D_{2S}R$ and MOR was detected by immunoblotting with A) anti-GFP and B) anti-D_2R.
